# EHMTI-0311. Effect of physical activity and hours of sleep on symptoms scores following migraine

**DOI:** 10.1186/1129-2377-15-S1-D42

**Published:** 2014-09-18

**Authors:** MT Moore, T Covassin

**Affiliations:** 1 School of Health and Human Performance, Northern Michigan University, Marquette, USA; 2 Kinesiology, Michigan State University, E. Lansing, USA

## Introduction

Physical activity and regular sleep are a common recommendation by all physicians to mitigate a myriad of disorders. Migraine treatment typically targets reduction of symptoms after onset of the migraine as their primary objective, yet little data exist comparing the symptom scores for medication choices.

## Aim

The purpose of this research is to determine how physical activity status, type of medication and hours of sleep effect the number/intensity of symptoms a migraineur reports.

## Methods

44 migraineurs reported hours of sleep and symptoms scores 0-6 on a Likert scale (0=no symptoms, 6 =max) for 22 symptoms associated with migraine at baseline, 24 hours, 48 hours and 7 days post migraine. Physical activity (meeting CDC minimum criteria) and type of medication [over the counter (OTC), prescription (Rx) OTC + Rx, None] were reported.

## Results

Repeated Measures ANOVA found there was no significant difference between migraineurs in hours of sleep (p=.698, F=.479) or physical activity status (p=.822, F=.304) at each measure, but a significant difference in total symptom scores by time (p<.001, F=18.507). Migraineur symptom scores peaked at 24 hours post migraine measurements. A one way ANOVA revealed significant differences between type of medication groups at 24 hours (p=.001, F=7.210) by treatment.

## Conclusions

The number of hours of sleep and physical activity status had no effect on the number or intensity of symptoms in migraineurs. It is important to determine outcomes of medications on symptom reduction for each patient to choose the best migraine management strategy.

No conflict of interest.

